# Inclusion Membrane Proteins of *Chlamydia trachomatis*: A Review with Emphasis on Biological Functions and Host Cell Modulation

**DOI:** 10.3390/microorganisms14071443

**Published:** 2026-06-30

**Authors:** Yujia Guo, Jie Xiao, Bingbing Su, Yufen Xiao, Lanhua Zhao

**Affiliations:** Institute of Pathogenic Biology, Hengyang Medical College, University of South China, Hengyang 421001, China; m18953094782@163.com (Y.G.); jiexiao0214@163.com (J.X.); su1613838660@outlook.com (B.S.); 17886912075@163.com (Y.X.)

**Keywords:** host–pathogen interactions, *Chlamydia trachomatis*, Inc proteins, functions

## Abstract

*Chlamydia trachomatis* is an obligate intracellular prokaryote. During the process of infecting host cells, *C. trachomatis* forms a unique inclusion structure, which serves as a critical niche for the survival and replication of *C. trachomatis* within host cells. The inclusion membrane is composed of various inclusion membrane proteins (Inc proteins), which play a pivotal role in the interaction between *C. trachomatis* and host cells. This review illustrates functional research progress of *C. trachomatis* Inc proteins and their molecular interactions with host cells, facilitating deeper understanding of chlamydial pathogenic mechanisms.

## 1. Introduction

*Chlamydia trachomatis* is an obligate intracellular human pathogen and one of the most prevalent etiological agents of bacterial sexually transmitted infections (STIs) worldwide, with an estimated 130 million new cases annually [1,2,3]. Coinfection of *Chlamydia* with other microbes may result in more severe inflammation and even tumor development [4,5].

*C. trachomatis* exhibits a unique biphasic life cycle. Extracellular infectious elementary bodies (EBs) invade host cells and are subsequently enclosed in membrane-bound vacuoles termed inclusions. Inside the inclusions, EBs differentiate into metabolically active reticulate bodies (RBs), the replicative form responsible for proliferation. Following proliferation, mature progeny EBs are released to initiate a new infection cycle [6].

The inclusion is a crucial site for the replication and biosynthesis of *Chlamydia*, providing a protected environment for the pathogen that enables it to evade the host’s immune clearance. In addition, *C. trachomatis* obtains nutrients from the host cell through the inclusion and expels metabolic waste into the host cell [7]. Inc proteins are a class of proteins localized to the inclusion membrane of *Chlamydia*. As structurally conserved proteins, Inc proteins carry an N-terminal type III secretion signal required for secretion from chlamydiae, plus a conserved hydrophobic domain with at least two transmembrane α-helices that anchors them to the inclusion membrane [8]. Inc proteins are exposed to the host cytoplasm and accumulate in kinase-rich microdomains [9,10]. These proteins also engage in intermolecular structural interactions within the family to achieve synergistic functions. Due to their essential involvement in chlamydial virulence, Inc proteins serve as ideal targets for drug development. Elucidating their functions and regulatory pathways facilitates the creation of innovative anti-chlamydial interventions and supports the prevention of sexually transmitted diseases [11,12]. Considering their critical roles in *C. trachomatis* pathogenesis, the present review systematically outlines recent advances in Inc protein studies, highlighting widespread molecular interactions between Inc proteins and host cells throughout *C. trachomatis* infection.

## 2. Mediate the Structural Stability of Inclusions and Reshape the Host Cytoskeleton

**Figure 1:**
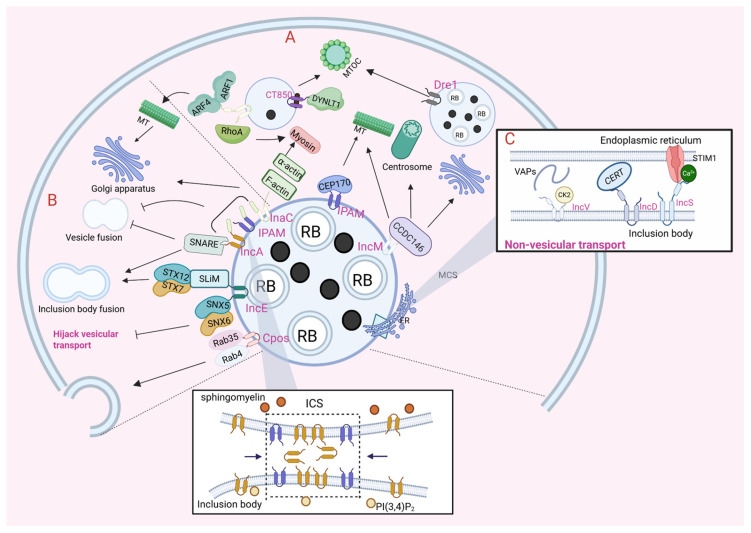
(**A**) Inc proteins drive host cytoskeletal remodeling for inclusion development and integrity. IPAM-CEP170 interaction stabilizes microtubules (MTs) at the inclusion surface. CT850 engages DYNLT1 to mediate inclusion trafficking toward the microtubule-organizing center (MTOC). InaC activates ARF1/ARF4 and RhoA signaling to regulate actin polymerization and myosin activity. IncM recruits CCDC146 to centrosomes and other host structures to reinforce inclusion stability. Sphingomyelin and PI(3,4)P_2_ accumulate at the inclusion contact site (ICS) (dashed box) to recruit IncA; trans IncA-IncA interactions amplify clustering for fusion initiation, while IPAM maintains inclusion structural integrity. Dre1 targets dynactin to reposition organelles around the inclusion and further enhance its structural integrity. RB, reticulate body. (**B**) Inc proteins hijack host vesicular trafficking and fusion machineries to support inclusion development and nutrient acquisition. IncA interacts with host soluble attachment protein receptor for N-ethylmaleimide-sensitive factor (SNARE) machinery via SNARE-like domains to mediate homotypic inclusion fusion and block harmful heterotypic vesicle fusion. IPAM and InaC also possess SNARE motifs to antagonize host vesicle fusion, while InaC additionally regulates Golgi architecture and actin cytoskeleton remodeling. CpoS recruits host Rab4 and Rab35 GTPases to intercept recycling vesicles, maintaining inclusion stability and supporting lipid acquisition from the Golgi apparatus. IncE binds the retromer components sorting nexin 5 (SNX5) and sorting nexin 6 (SNX6), and recruits STX7/STX12 via distinct short linear interaction motifs (SLiMs), hijacking retrograde vesicular transport to disrupt host trafficking and facilitate nutrient uptake. (**C**) Inc proteins mediate endoplasmic reticulum (ER)–inclusion membrane contact sites (MCSs) for non-vesicular transport and host organelle manipulation. IncV interacts with ER-resident VAP proteins to stabilize the MCS. IncD recruits the ceramide transfer protein (CERT) to the MCS, enabling non-vesicular ceramide transport from the ER to the inclusion. IncS engages ER-localized stromal interaction molecule 1 (STIM1) to modulate calcium (Ca^2+^) signaling at the contact site.

As the inclusion continues to expand, *C. trachomatis* ingeniously utilizes four cytoskeletal components that function to sustain inclusion stability and architecture: microtubules (MTs), actin, intermediate filaments (IFs), and septin proteins [13,14]. IFs provide stability to the expanding inclusion, while MTs are progressively organized on the surface of the inclusion and assemble into interconnected scaffolds and nest-like superstructures. Septin proteins are cytoskeletal proteins that directly interact with membranes, acting as scaffolds to recruit proteins to cellular locations and as structural diffusion barriers [15]. Actin is closely related to the invasion and release processes of *C. trachomatis* [16]. At various developmental stages of *C. trachomatis*, Inc proteins reshape microfilaments and MTs of the host cell cytoskeleton [17]. Hijacking the host MT cytoskeleton must be an important component of *C. trachomatis* infection (Figure 1A). InaC (CT813), IPAM (CT223), IncM (CT288), and CT850 function sequentially to modulate microtubule and actin networks, mediate inclusion transport to the microtubule-organizing center (MTOC), and maintain inclusion morphology [18,19,20]. IncA serves as the central fusogen that drives homotypic fusion by forming trans-interactions at specialized inclusion contact sites (ICSs), which is tightly regulated by PI(3,4)P_2_ and sphingomyelin [21]. Dre1 (CT192/CTL0444) further enhances *C. trachomatis* fitness by targeting host dynactin to reposition key organelles around the inclusion and stabilize its structure [22].

InaC is mainly responsible for regulating the stability of the cytoskeleton during *C. trachomatis* infection [19]. By comparing the characteristics of InaC mutant and wild-type strains, Kokes et al. found that InaC is involved in the accumulation of F-actin around inclusions [23]. InaC also recruits ADP-ribosylation factor 1 (ARF1) and ARF4 to the inclusion membrane and modulates their activation; the activated ARF1 governs the formation of post-translationally modified MTs, coordinates the assembly of microfilaments around the inclusion, and thereby finely regulates the distribution of the Golgi apparatus around the inclusion [24,25]. In addition to stabilizing MTs, InaC regulates the stability of the cytoskeleton by controlling the actin scaffold to support the integrity of the envelope during infection. InaC recruits RhoA to promote the formation of the actin scaffold around the inclusion, and activated RhoA further drives actin polymerization at the inclusion periphery [13,24]. *C. trachomatis* also hijacks α-actin to cross-link these scaffolds and stabilize the inclusion structure. α-actin is recruited to the inclusion membrane in an InaC-dependent manner, which reinforces the expanding survival niche for *C. trachomatis* and enables tight binding to the actin scaffold of the inclusion [26]. In summary, InaC guides the dynamics of actin and post-translationally modified MT scaffolds by mediating the crosstalk between cytoskeleton elements and GTPases of RhoA and ARF1.

The Inc protein IPAM and the host centrosome protein CEP170 are involved in the assembly of MTs around the *C. trachomatis* inclusion [18]. IPAM is located in the inclusion microdomain near the centrosome. It interacts with the pericentriolar material (PCM) on the centrosome by virtue of its primary structure similar to centrosome and MT-associated proteins, and thus interferes with MT assembly. IPAM locally hijacks the host MT-organizing activity by recruiting and stimulating CEP170. Knocking down CEP170 in *C. trachomatis*-infected cells results in the reduction in the MT scaffold and disappearance of the MT nest, leading to a characteristic rounded cell morphology. Meanwhile, CEP170 depletion triggers defects in MT organization during MT regrowth and causes aberrant inclusion morphology [17]. Therefore, CEP170 is critical for *C. trachomatis* to govern host MT assembly, drive inclusion morphogenesis, and maintain bacterial infectivity [17,27].

IncM localizes to inclusion microdomains. It interferes with the division process of host cells [28], affects the localization of the centrosome [29], and changes the distribution of the Golgi apparatus around the inclusion [20], thus affecting the morphology and stability of the inclusion. Almeida F. et al. reported that IncM targets the centrosome, modulates centrosome positioning and remodels the MT cytoskeleton. A novel interaction between IncM and host cell protein CCDC146 has been identified. CCDC146 is recruited to the periphery of the inclusion membrane, suggesting a potential key role during the host cell infection by *C. trachomatis* [30]. Collectively, the molecular mechanism underlying IncM-mediated cytoskeleton regulation remains poorly understood. Importantly, the direct functional relationship between IncM and CCDC146 has not been clarified in existing studies.

It is worth noting that the *C. trachomatis* inclusion membrane protein CT850 interacts with dynein light chain 1 (DYNLT1), mediating the transport process of the inclusion to the microtubule-organizing center. Research shows that the deletion of DYNLT1 disrupts the characteristic connection between the inclusion membrane and the centrosome, reducing the association between the *C. trachomatis* inclusion and the MTOC. On the *C. trachomatis* inclusion membrane, DYNLT1, which is known to interact with dynein and MTs, aggregates to form concentrated foci mediated by CT850 [31]. In summary, the interaction between CT850 and DYNLT1 contributes to the correct positioning of inclusions at the MTOC. IncA acts as the core fusogenic effector that mediates inclusion homotypic fusion by trans-interacting and clustering at specialized ICSs; this process is spatially controlled by PI(3,4)P_2_ and sphingomyelin, and further supported by IPAM to maintain inclusion structural stability [21]. Recently, the Inc protein Dre1 has been shown to target host dynactin at MTOCs, thereby repositioning the centrosome, Golgi, and other organelles around the inclusion to stabilize inclusion structure and promote *C. trachomatis* fitness [22]. Overall, the precise molecular mechanisms underlying effector–target binding and pathway crosstalk remain elusive, and relevant clinical validations are pending further investigation.

## 3. Hijack the Host Cell’s Vesicular Transport

When interacting with other pathways within host cells, the inclusions of *C. trachomatis* deviate from the normal lysosomal pathway and obtain the nutrients required for growth and replication from the host cells. *C. trachomatis* exploits its own Inc proteins to hijack host vesicular and non-vesicular transport machineries. By modulating intracellular trafficking and membrane fusion, the pathogen sequesters essential nutrients from the host cytoplasm to sustain its survival and replication, and supports inclusion maturation [32,33,34] (Figure 1B). Functional differentiation among distinct Inc proteins drives extensive remodeling of host intracellular trafficking networks, inhibition of host innate restriction against infection, and sustained survival and proliferation of *C. trachomatis* inclusions. Distinct members of the *C. trachomatis* Inc protein repertoire, such as IncA, CpoS and IncE (CT116), mediate disparate regulatory effects on the host vesicular trafficking machinery. IncA structurally mimics host soluble N-ethylmaleimide-sensitive factor (NSF) attachment protein receptor (SNARE) proteins to hijack host membrane fusion machinery [35,36]; CpoS remodels RAB GTPase-dependent vesicular trafficking to reroute nutrient-containing host vesicles toward inclusions [33,37]; and IncE engages sorting nexin 5 (SNX5), sorting nexin 6 (SNX6), and STX7 and STX12 through distinct short linear interaction motifs (SLiMs) to subvert retrograde vesicular transport and facilitate inclusion fusion [38].

*C. trachomatis* is capable of obtaining nutrients by hijacking the vesicular transport of host cells. SNARE proteins, together with small guanosine triphosphate (GTP)-binding proteins including ADP-ribosylation factors (ARFs) and RAB GTPases, are key regulators of membrane vesicle trafficking and fusion in eukaryotic cells [33]. SNAREs are key components of the intracellular fusion machinery, where vesicular SNAREs bind to their corresponding target membrane SNAREs to drive membrane fusion. ARFs are small GTPases that play a crucial role in regulating vesicular transport by recruiting the coat proteins required for vesicle formation [39]. RAB GTPases are localized to different organelles, are the main regulators of vesicular transport in eukaryotes, and regulate vesicle budding, transport, docking, and fusion [33,34,40]. Some of them (RAB 1, 4, 6, 8, 10, 11, 14, 34, 35, 39a, 39b) have been reported to be localized on or near the inclusion membrane of *C. trachomatis* [33,41,42,43]. In *C. trachomatis*, several inclusion membrane proteins such as IncA, IPAM and InaC possess SNARE motifs that mimic SNARE proteins and inhibit harmful vesicular fusion [35,36]. For instance, IncA possess two SNARE-like coiled-coil domains, SLD-1 and SLD-2, on the inclusion membrane, which mediate homotypic inclusion fusion and block SNARE-dependent fusion, with each domain alone exerting this inhibition [44,45].

The recruitment of Rab proteins is considered to reflect the ability of bacteria to capture host vesicles and thus obtain lipids. Inc proteins play an important role in the recruitment of RAB GTPases [46,47]. Among them, CpoS functions as one of the key regulators of host cell vesicular transport in *C. trachomatis*-infected cells. It targets various RAB GTPases and their corresponding effector proteins to the inclusions, intercepts host vesicles from the recycling pathway, and redirects Rab-containing vesicles to the inclusions, thereby regulating the transport of transferrin and mannose-6-phosphate receptors [33]. Furthermore, the latest genome-wide screening evidence reveals that CpoS preserves the stability of *C. trachomatis* inclusions by regulating Rab-dependent vesicular transport of Golgi-originated sphingolipids, while the ceramide transfer protein (CERT)-mediated non-vesicular ceramide transport pathway remains unperturbed [37]. Meier K. et al. also contend that loss of CpoS diminishes ceramide acquisition by *C. trachomatis* inclusions [46].

Beyond modulating Rab GTPase-dependent vesicular trafficking, Inc proteins also hijack host retrograde transport machineries to facilitate intracellular survival. SNXs are important components of the retrograde transport complex. It contains a PX (phox homology) domain, which mediates the recycling of endosomal substances to the plasma membrane or the trans-Golgi network (TGN) [27]. It is worth noting that various sorting nexins will accumulate in the specific cellular structure of the inclusion. Among them, the inclusion membrane protein IncE binds to the PX domain of the sorting nexin SNX5/6 and recruit these components of the retrograde transport complex to the inclusions [48]. This inhibits host retrograde vesicular trafficking and attenuates host cellular restriction to *C. trachomatis* infection, thereby promoting pathogen survival and replication inside host cells and elevating infectious progeny production [49]. For example, IncE competes with the cation-independent mannose-6-phosphate receptor (CI-M6PR) for binding to SNX5, inhibits the interaction between SNX5 and CI-M6PR, and further affects the binding of CI-M6PR cargo to the endosomal subdomain containing retromer, resulting in disorders in the transport and recycling processes of lysosomal enzymes [34,50,51]. IncE possesses two distinct SLiMs at its cytosolic C terminus. The proximal SLiM mimics an R-SNARE fragment to recruit STX7/STX12-bearing vesicles, while the distal SLiM recognizes the cargo-binding site of SNX5/SNX6 and recruits SNX6-associated vesicles to the inclusion [38]. This action underscores the sophisticated manipulation of host vesicular transport systems by Inc proteins.

## 4. Regulate Host Cell’s Non-Vesicular Transport

The inclusion of *C. trachomatis* not only manipulates the vesicular transport between host organelles but also establishes direct membrane contact sites (MCSs) with organelles such as the endoplasmic reticulum (ER) [52]. The formation of MCSs is regarded as a mechanism by which intracellular pathogens hijack cellular resources and establish their own replication microenvironment (Figure 1C). Pathogens can utilize MCSs [53]. Inc proteins including IncD, IncV and IncS exert pivotal functions in MCSs, sustaining MCS stability and modulating organelle functions and interorganellar interactions.

The ER integral proteins VAPA and VAPB (collectively referred to as VAPs) are common components of the MCSs formed between the ER and various organelles [54]. The ER-resident CERT drives non-vesicular transport of sphingomyelin precursor ceramide toward the trans-Golgi network. At ER–inclusion MCSs enriched with ER-localized VAPA/B, *C. trachomatis* secretes effector IncD (CT115) to bind CERT, assemble the IncD/CERT/VAP complex, and recruit CERT to such MCSs. By hijacking the CERT-VAP machinery, the pathogen reroutes ceramide away from canonical ER-to-Golgi trafficking and mediates non-vesicular ceramide shuttling from the ER into inclusions to secure sphingomyelin for its intracellular survival [34,54,55,56]. At the MCS formed between the *C. trachomatis* inclusion membrane and VAPA/B-containing ER tubules, IncD binds CERT, recruits the protein to this contact site, and facilitates non-vesicular ceramide delivery from the ER to inclusions for sphingomyelin synthesis [53,57,58]. IncD may interact with as-yet-unidentified host or bacterial factors that localize to the inclusion membrane. Further structural and functional characterization of IncD is required to address these questions. IncV (CT005) plays a structural role in mediating the formation of MCS. IncV inserted into the inclusion membrane is one of the main molecular bridges promoting the formation of ER–inclusion MCSs. The cytoplasmic C-terminal tail of IncV contains two FFAT motifs, which synergistically mediate the interaction between IncV and VAPA/B, thereby promoting the formation of ER–inclusion MCSs [54]. Overexpression of IncV is sufficient to bring the ER closer to the membrane containing IncV. Through its C-terminal region containing three CK2 phosphorylation motifs, IncV recruits CK2 to the inclusions, and the assembly of the IncV-VAP complex is regulated by the post-translational phosphorylation of the host kinase CK2 [59]. However, the deletion of IncV only partially reduces the association between VAP and the inclusions and does not inhibit the formation of ER–inclusion MCSs, indicating that IncV may have functional redundancy with other factors involved in establishing MCS [54].

In addition to CERT, stromal interaction molecule 1 (STIM1) is also recruited to the MCS. The ER calcium (Ca^2+^) sensor STIM1 can maintain Ca^2+^ homeostasis through store-operated calcium entry (SOCE) [60]. The ER–Golgi MCS and the ER–inclusion MCS are, respectively, related to the non-vesicular lipid transport from the ER to the Golgi apparatus and Ca^2+^ homeostasis [52]. Therefore, *C. trachomatis* encodes an Inc protein similar to Orai1 to participate in the recruitment of STIM1, namely IncS. IncS is a new component of the ER–inclusion MCS of *C. trachomatis*. It directly interacts with the cytoplasmic domain of the previously identified host component STIM1 at the ER–inclusion MCS [61]. This specific interaction recruits STIM1 to the ER–inclusion MCS and promotes their co-localization [62].

## 5. Modulate Host Cell Survival

**Figure 2:**
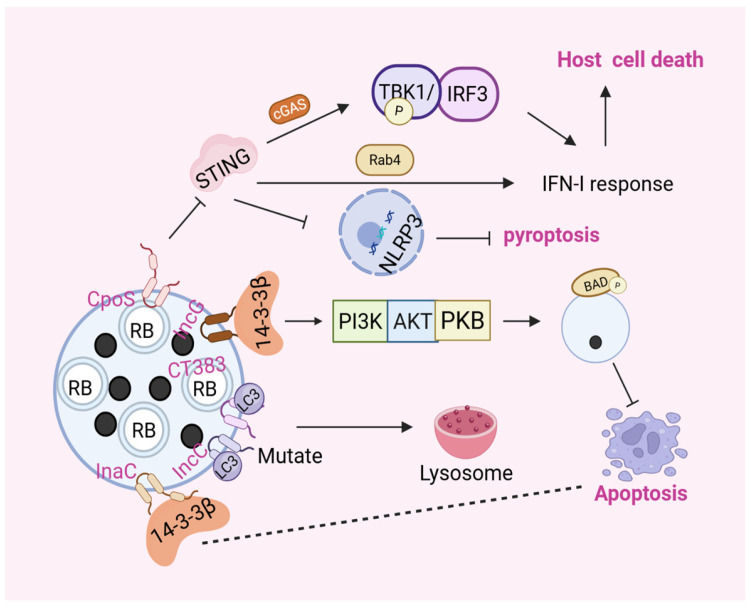
Inc proteins manipulate multiple host cell death pathways to sustain intracellular infection. CpoS interacts with Rab4 to target ER-localized stimulator of interferon genes (STING), thereby suppressing the cGAS-STING-TBK1/IRF3 axis and NLRP3 inflammasome activation to block premature host pyroptosis and type I interferon responses during early infection. IncG binds host 14-3-3β and activates the PI3K/AKT signaling cascade, which maintains BAD phosphorylation to inhibit the intrinsic apoptotic pathway. IncC and CT383 preserve inclusion membrane integrity to avoid LC3-mediated autolysosomal degradation and prevent premature inclusion lysis and apoptosis. The interaction between InaC and 14-3-3β represents a potential uncharacterized link to apoptotic modulation during infection.

The completion of the replication cycle of *C. trachomatis* entirely depends on the host environment [63,64]. To maintain this process, it promotes the survival of host cells by inhibiting apoptosis [65,66]. Studies have demonstrated that the absence of specific Inc proteins including CpoS, IncC and CT383 causes premature lysis of *C. trachomatis* inclusions, triggering autolysosomal recognition, activation of endogenous apoptosis, and premature arrest of the *C. trachomatis* developmental cycle [67]. In addition to apoptosis regulation, Inc proteins broadly remodel multiple host cell death programs, including pyroptosis, apoptosis, and autophagy. These functionally distinct Inc proteins work in concert to govern the central signaling axis reliant on stimulator of interferon genes (STING), the key cytosolic DNA sensor adaptor. Via stage-specific tuning of host cell death programs, these proteins promote sustained intracellular colonization of *C. trachomatis* (Figure 2).

To avoid excessive premature pyroptosis in early infection, CpoS serves as a critical anti-pyroptotic effector. CpoS interacts with the GTPase Rab4 to target ER-localized STING, thereby suppressing type I interferon responses and pro-death signaling to block early host cell death [68]. CpoS loss-of-function mutations trigger STING activation and its translocation from the ER to perinuclear vesicles, which activates the cGAS/STING/TBK1/IRF3 pathway to induce robust type I interferon responses. As a central signaling hub, STING bridges interferon signaling and inflammasome activation, and the cGAS–STING pathway tightly restrains NLRP3 inflammasome assembly. Upon full activation, the NLRP3 inflammasome triggers caspase-1 maturation; activated caspase-1 subsequently cleaves gasdermin D (GSDMD), which oligomerizes to form plasma membrane pores and ultimately executes host pyroptosis [67,68,69]. Notably, direct evidence verifying that CpoS restrains pyroptosis specifically through the cGAS-STING axis remains insufficient [68]. Independently of inflammasome signaling, STING binds the ER calcium pump SERCA2 to disrupt ER calcium homeostasis and trigger cell death, and pharmacological SERCA inhibition alleviates the cytotoxicity caused by CpoS-deficient *C. trachomatis* infection [68,70]. Overall, STING integrates multiple cell fate regulatory networks and is differentially manipulated by distinct Inc proteins throughout infection.

In contrast to the early pro-survival function of CpoS, the Inc protein GarD mediates late-stage STING-dependent host cell lysis to support chlamydial proliferation. During the late developmental stage of *C. trachomatis* inclusions, GarD facilitates STING translocation from the ER to the Golgi apparatus, which initiates host cell lysis [71,72]. GarD overexpression further disrupts inclusion membrane integrity and aggravates host cell death, highlighting its pro-lytic role in late infection [71].

Multiple Inc proteins synergistically inhibit host apoptosis to stabilize intracellular *C. trachomatis* survival. IncC and CT383 preserve inclusion membrane stability to prevent premature inclusion lysis and host cell death. Defective IncC or CT383 function results in LC3 labeling of impaired inclusions, followed by autolysosomal degradation and endogenous host apoptosis [67,68]. This dynamic interplay between IncC/CT383 and host autophagy is pivotal for maintaining intracellular infection homeostasis, although the detailed mechanism linking their anti-apoptotic function to STING signaling remains uncharacterized. In addition to membrane-stabilizing anti-apoptotic mechanisms, IncG (CT118) regulates host apoptosis by binding the host adaptor protein 14-3-3β [67,73]. The IncG-14-3-3β interaction activates the PI3K/AKT signaling cascade, which maintains phosphorylation of the pro-apoptotic factor BAD and suppresses its pro-death activity. Phosphorylated BAD is sequestered at *C. trachomatis* inclusions, preventing mitochondrial cytochrome c release and inhibiting host intrinsic apoptosis [73,74]. Studies related to immunoprecipitation reactions and mass spectrometry analysis have revealed the binding of the ε, η, ζ, γ, θ, and β subtypes of 14-3-3β to InaC [23]. To date, no available evidence has functionally linked the interaction between InaC and 14-3-3β to host apoptotic modulation; nevertheless, this physical association provides a promising research direction, and subsequent investigations may uncover unrecognized roles of InaC in *C. trachomatis*-governed apoptotic pathways.

Some studies predict that factors such as CT227, CT058, CrpA, and CT449 may be related to the regulation of apoptosis [27]. However, currently, there is no specific literature elaborating in detail. Regardless of whether *C. trachomatis* promotes or inhibits host cell apoptosis, these mechanisms all represent evolutionary adaptations that enable its survival within host cells.

## 6. Orchestrate the Host Cell Immune Response

**Figure 3:**
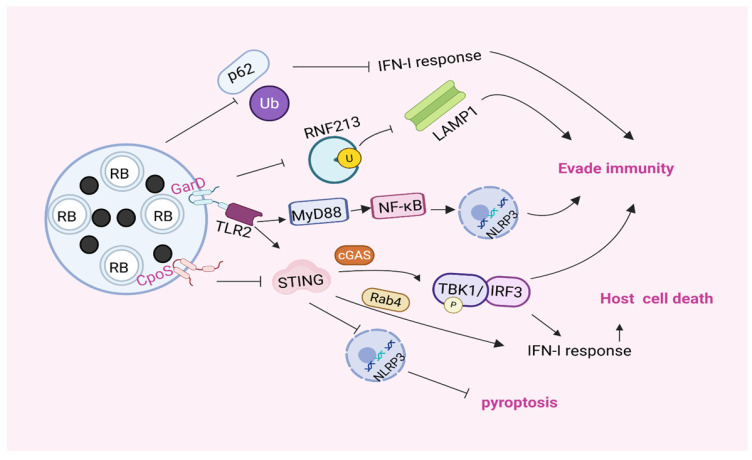
Inc proteins mediate immune evasion and host cell fate control through multiple signaling axes. GarD blocks RNF213-dependent ubiquitination of the inclusion to evade IFN-γ-mediated autophagic clearance and lysosome-associated membrane protein 1 (LAMP1) targeting. GarD activates TLR2-MyD88-NF-κB signaling to trigger NLRP3 inflammasome activation, counteracting neutrophil defense. CpoS inhibits STING-dependent type I IFN responses and pyroptosis by interacting with Rab4, limiting early host immune surveillance.

Inc proteins actively mediate immune evasion of *C. trachomatis* through distinct molecular mechanisms to counteract host immune surveillance and support intracellular bacterial replication and persistent infection (Figure 3). GarD interferes with IFN-γ-triggered cell-autonomous immunity and neutrophil-mediated defense, while CpoS represses STING-dependent type I interferon signaling. Distinct molecular mechanisms empower *C. trachomatis* to evade host immune attack and develop persistent infection.

The γ-resistance determinant GarD (CT135) of *C. trachomatis* Inc protects the inclusion from the attack of cellular autonomous immunity and is a genuine immune evasion factor [75]. A key ubiquitin E3 ligase, RNF213, effectively labels the inclusion and mediates the downstream clearance process. GarD prevents RNF213 from targeting the inclusion, thereby blocking the IFN-γ-dependent ubiquitin-mediated inclusion labeling process, preventing the binding of lysosome-associated membrane protein 1 (LAMP1) to the inclusion, protecting the inclusion from ubiquitin-dependent destruction, and enabling it to evade the cellular autonomous immunity activated by IFN-γ [75,76,77]. As a crucial Inc protein, GarD acts through the above mechanism to inhibit the recruitment of ubiquitin and p62/SQSTM to the inclusion surface. This immune evasion strategy is conserved across human and nonhuman primate hosts, and a loss of GarD severely attenuates *C. trachomatis* growth and survival both in vitro and in vivo [78]. These findings highlight GarD as an essential virulence determinant that allows *C. trachomatis* inclusions to evade host degradation and establish productive infection.

Moreover, GarD specifically activates the NLRP3 inflammasome in neutrophils during the infection of *C. trachomatis*, thus evading the host defense mediated by neutrophils [71,79]. CpoS (CT229) is a critical suppressor of host cellular immune surveillance and regulates the IFN signaling pathway through interaction with Rab proteins. The CpoS-Rab interaction effectively suppresses the type I interferon response mediated by STING, and weakens the host’s defense mechanism by deeply participating in blocking the host immune signaling pathway [46,77,80]. Overall, these mechanisms reflect the sophisticated strategy adopted by *C. trachomatis* to manipulate host immune responses for intracellular survival and proliferation.

## 7. Coordinate the Production and Release of EBs

**Figure 4:**
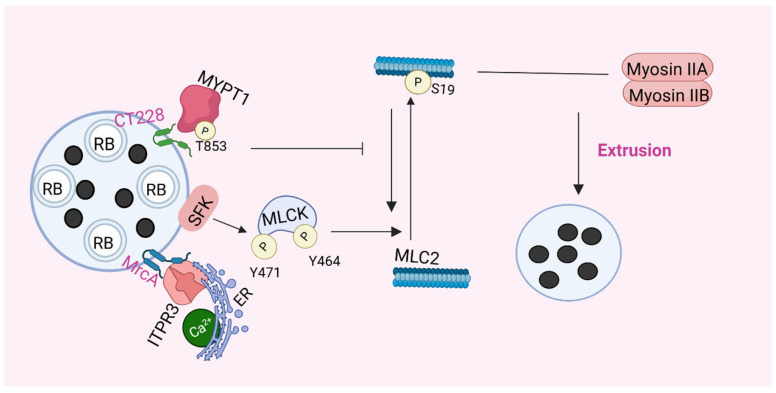
A model of *C. trachomatis* inclusion extrusion. CT228 recruits myosin phosphatase targeting subunit 1 (MYPT1) to inclusion-associated microdomains, preventing premature MYPT1 phosphorylation and myosin light chain 2 (MLC2) activation to block early inclusion extrusion. MrcA binds and recruits the ER-resident Ca^2+^ channel inositol 1,4,5-trisphosphate receptor type 3 (ITPR3) to the inclusion–ER interface, triggering Ca^2+^ release to elevate cytoplasmic Ca^2+^ levels. Elevated Ca^2+^ activates myosin light chain kinase (MLCK) and promotes inhibitory phosphorylation of MYPT1, sustaining MLC2 phosphorylation and myosin motor activity. Phosphorylated MLC2 engages myosin IIA and myosin IIB to form the contractile motor complex that drives inclusion extrusion and elementary body (EB) release.

After completion of the *C. trachomatis* replication cycle, EBs are released from host cells via two routes: cell lysis (rupture of both the inclusions and host cells) or exocytosis (inclusions enveloped by the host plasma membrane are expelled from the host cells) [27,62]. Extrusion represents an active release process dependent on actin cytoskeleton remodeling and functional Inc proteins [81], and vesicles formed during extrusion protect extracellular bacteria to improve the survival of *C. trachomatis* [82]. CT228 and MrcA, two representative Inc proteins, orchestrate inclusion extrusion and egress of *C. trachomatis*. By interacting with host molecules, they regulate myosin light chain 2 (MLC2) phosphorylation, calcium homeostasis and cytoskeleton dynamics (Figure 4). Notably, GarD mediates bacterial release via the STING-dependent pathway.

CT228 recruits myosin phosphatase targeting subunit 1 (MYPT1) to the microdomains around the inclusion and regulates the extrusion and release of EBs [83]. Research by Lutter E.I. et al. indicates that in the early stage of *C. trachomatis* infection, the interaction between CT228 and MYPT1 prevents the phosphorylation of MYPT1, and MLC2 is dephosphorylated and inactivated, thus preventing the premature extrusion of inclusions. In the later stage of the developmental cycle, phosphorylated but inactive MYPT1 enriches in the microdomains, which is conducive to maintaining phosphorylated and active myosin light chain 2 (MLC2) and myosin light chain kinase (MLCK). MLC2 interacts with myosin IIA and myosin IIB to form an active myosin motor complex, which promotes the expulsion of inclusions and initiates a new round of the infection cycle [83,84].

Ca^2+^ signaling plays a pivotal role in the regulation of extrusion. The combined effect of increased intracellular Ca^2+^ leads to the activation of MLCK and the inhibition of antagonistic myosin phosphatase, favoring phosphorylation, which promotes extrusion [84]. Inositol 1,4,5-trisphosphate receptor type 3 (ITPR3) is an ER cation channel that conducts Ca^2+^ and is involved in calcium signaling [85]. STIM1 controls the intracellular Ca^2+^ level and is crucial for regulating store-operated calcium entry (SOCE) [85,86]. Therefore, both the calcium channel ITPR3 and the Ca^2+^ sensor STIM1 are of great importance during the process of exocytosis formation. The Inc protein MrcA binds to ITPR3 to regulate the calcium level, thereby promoting the extrusion and release [87]. MrcA recruits ITPR3 to the microdomain, where it regulates the release of Ca^2+^ from the reservoir by interacting with STIM1 and ITPR3, increasing the concentration of Ca^2+^ in the cytoplasm. The increase in the Ca^2+^ concentration not only promotes the phosphorylation of MLC2 but also activates Rho kinase, which in turn phosphorylates MYPT1, thus maintaining the activity of the myosin motor complex to facilitate the exocytosis process of *C. trachomatis*. Loss of MrcA function reduces the recruitment of ITPR3, which consequently restricts inclusion extrusion and egress [84]. IncS also specifically interacts with and recruits STIM1, which contributes to maintaining Ca^2+^ homeostasis [56].

Furthermore, InaC-dependent recruitment of F-actin to the inclusion membrane may also contribute to optimal extrusion [81], and IPAM is also considered a foci of extrusion [84]. GarD exits the bacterium via lysis during late stages of the developmental cycle in a STING-dependent manner [71].

## 8. Summary and Prospect

Benefiting from synergistic advances in bacterial genetics, structural biology and cell biology, substantial progress has been made in the functional characterization of Inc proteins. Table 1 summarizes known Inc proteins of *C. trachomatis*, highlighting distinct differences in their expression cycles, target host molecules of action, and main regulatory or functional roles in host cells during the bacterial intracellular developmental cycle.

However, several unresolved bottlenecks impede precise dissection of molecular cascades within Inc-centered regulatory networks and restrict comprehensive understanding of Inc-mediated host–pathogen interaction mechanisms. Firstly, most existing studies concentrate on the independent functions of individual Inc family proteins, while synergistic and antagonistic crosstalk among distinct Inc proteins remains barely explored. Second, the scarcity of in vivo experimental data on the Inc protein family severely limits the translational potential of fundamental mechanistic research on *C. trachomatis*, as most relevant observations are derived from simplified in vitro culture models that cannot recapitulate authentic tissue microenvironments of human infection. Finally, well-documented chlamydia–host metabolic crosstalk, key molecules governing pathogen developmental shifts, intracellular signal sensing and host immune responses remain unknown, and Inc proteins acting as central linkers of these processes are essential to decipher such complex pathogenic mechanisms.

In terms of technical methodologies, conventional biochemical strategies, including co-immunoprecipitation, affinity purification and site-directed mutagenesis, have been widely applied to verify interactions among *C. trachomatis* Inc proteins, bacterial effectors and host cellular factors. However, these traditional approaches fail to resolve the precise binding interfaces, interaction affinity and dynamic conformational remodeling of protein complexes during *C. trachomatis* infection. Cryo-electron microscopy (cryo-EM) overcomes these technical barriers and enables high-resolution structural dissection of Inc-centered supramolecular assemblies. Future cryo-EM-based investigations will facilitate the delineation of the spatial architecture of inclusion membrane–effector–host complexes, the identification of key binding residues and interfacial domains, and the comprehensive characterization of dynamic interaction landscapes throughout the entire *C. trachomatis* infection cycle.

Future research frameworks can integrate cryo-EM-based structural biology with state-of-the-art technologies, including single-cell RNA sequencing, quantitative proteomics and metagenomic next-generation sequencing (mNGS), together with optimized genetic manipulation systems and physiologically relevant infection models based on primary cells and organoids [101,102]. This multi-dimensional analytical platform will substantially advance the mechanistic understanding of the intricate pathogenic networks of *C. trachomatis*. Integrated structural and functional profiling of diverse Inc proteins will further elucidate their synergistic roles in regulating host cytoskeleton organization and centrosome homeostasis.

Collectively, while current studies have mapped basic functions of Inc proteins, integrated structural, multi-omics and physiologically authentic infection models are urgently required to address outstanding research gaps and fully decode the sophisticated Inc-dependent pathogenic mechanisms of *C. trachomatis*.

## Figures and Tables

**Table 1:** Known Inc proteins of *Chlamydia trachomatis*, their expression cycles, target molecules of action, and main functions in host cells.

Inc Protein			Expression Stage	Target Molecule in Host Cell	Known Functions
Strain D/UW3	Strain L2/434	General			
CT005 [88]	CTL0260	IncV	Early	VAPA/B	contribute to the formation of MCSs; mediate non-vesicular lipid uptake; tether the ER to the pathogen-containing vacuole [54,89]
CT006 [32]	CTL0261			GFP, lipid droplets (LDs) isoforms of 14-3-3β	correlate with the formation, function and organelle interaction of LDs; inhibit apoptosis [32]
CT101 [84]	CTL0356	MrcA	Metaphase, 8-12 hpi	ITPR3, STIMI	regulate extrusion [9,85]
CT115 [53]	CTL0370	IncD	Early	CERT	contribute to the formation of MCSs; mediate non-vesicular lipid uptake [53,58,90]
CT116 [50]	CTL0371	IncE	Early	SNXs 7	suppress retromer-mediated transport; reprogram vesicle trafficking [38,68]
CT117 [91]	CTL0372	IncF	Early	APEX2, VAMP3	mediate Inc-Inc interactions [3,91]
CT118 [73]	CTL0373	IncG	Early, 2hpi	14-3-3β, VAMP3	associate with LDs; inhibit apoptosis [73,91]
CT119 [35]	CTL0374	IncA	Metaphase, Transcribed 10–12 hpi	VAMP3/7/8 SNARE	promote homotypic fusion of inclusions; interfere with host cell vesicular trafficking; inhibit endocytic membrane fusion [36,91,92]
CT135 [75]	CTL0390	GarD	8hpi	RNF213 STING	shield inclusions from cellular autophagy; mediate host cell lysis and bacterial exit; block ubiquitin/p62 recruitment to evade IFN-γ-triggered immunity; facilitate chlamydial growth in primates in vitro and in vivo [71,76,78]
CT147 [61]	CTL0402	IncS	1 hpi, Entire developmental cycle expression	STIM1	form MCSs; maintain calcium homeostasis; modulate extrusion [61]
CT192 [22]	CTL0444	Dre1	Mid-stage	Dynactin complex	recruit dynactin to inclusion; reposition host centrosome and Golgi; facilitate inclusion homotypic fusion; improve chlamydial progeny production [22]
CT222 [9]	CTL0475		Metaphase, 8-12 hpi		mediate Inc-Inc interactions [9]
CT223 [17]	CTL0476	IPAM	Metaphase, 8 hpi	CEP170	manipulate host cell MTs; inhibit vesicle fusion and host cell transport; block host cell cytokinesis [17]
CT224 [88]	CTL0477		Metaphase	TRAF7 (Tumor necrosis factor (TNF) receptor-related factors)	inhibit host cell cytokinesis
CT225 [18]	CTL0477A		Metaphase		inhibit host cell cytokinesis [18]
CT226 [93]	CTL0478			leucine rich repeat Flightless-1 (LRRF1)	govern the localization of FLI1 and LRRF1 to the inclusion
CT228 [83]	CTL0480			MYP1	prevent *C. trachomatis* compression; modulate MYPT1 recruitment, bacterial excretion and infection persistence [30,94]
CT229 [47]	CTL0481	CpoS	Early, 1 hpi	RABs Rab4, 35, STING	maintain envelope stability and control host cell death; modulate host vesicle transport and non-vesicular trafficking; inhibit host cell death and immune signaling pathways [33,46,67,68]
CT232 [95]	CTL0484	IncB	2 hpi	Snapin (SNARE-associated protein)	establish protective Th1 response to eliminate *C. trachomatis*-infected host cells [9]
CT233 [95]	CTL0485	IncC	2 hpi	ARF1,4	regulate envelope stability; establish a protective Th1 cell response to eliminate *C. trachomatis*-infected host cells [67]
CT288 [30]	CTL0540	IncM		CCDC146	interfere with host cell cytokinesis, centrosome positioning and Golgi distribution; maintain the stability of pathogen-containing vacuole [30,96]
CT383 [7]	CTL0639				regulation of envelope stability, inhibit apoptosis
CT440 [97]	CTL0699		12 hpi	Ret finger protein (RFP)	may participate in the *C. trachomatis* interactions with host cells [97]
CT442 [98]	CTL0701	CrpA		MHC-I, VAMP3	activate the adaptive immune response [91]
CT529 [99]		Cap1		MHC-I	activate the adaptive immune response [99,100]
CT618 [100]	CTL0882				associate with LDs
CT813 [19]	CTL0184	InaC	Metaphase, Earlyto 12 hpi	14-3-3 proteins, ARF1/4, CREB3 VAMP3/7/8, RhoA	stabilize actin scaffolds and MT scaffolds; act as cytoskeletal core stabilizer; manipulate MTs and induces actin assembly and Golgi redistribution around the inclusion; elicit the host immune response; inhibit apoptosis [19,25,91]
CT850 [31]	CTL0223		Metaphase, 2 hpi	DYNLT1	promote appropriate positioning of the inclusion at the MTOC [9]

## Data Availability

No new data were created or analyzed in this study. Data sharing is not applicable to this article.
